# PET imaging of platelet derived growth factor receptor β in lung fibrosis

**DOI:** 10.1186/s41181-025-00366-3

**Published:** 2025-07-15

**Authors:** Olivia Wegrzyniak, Francesco Lechi, Johanna Rokka, Bogdan Mitran, Bo Zhang, Ulrika Thelander, John Löfblom, Fredrik Y. Frejd, Olle Korsgren, Gaetano Perchiazzi, Jonas Eriksson, Olof Eriksson

**Affiliations:** 1 Science for Life Laboratory, Department of Medicinal Chemistry, Uppsala University, Dag Hammarskjölds väg 14C, 3tr, 751 83 Uppsala, Sweden; 2 Antaros Tracer AB, Uppsala, Sweden; 3 Department of Immunology, Genetics and Pathology, Uppsala University, Uppsala, Sweden; 4 Department of Protein Science, Division of Protein Engineering, KTH Royal Institute of Technology, Stockholm, Sweden; 5 Affibody AB, Solna, Sweden; 6 Hedenstierna Laboratory, Department of Surgical Sciences, Uppsala University, Uppsala, Sweden; 7 Anesthesia, Operation and Intensive Care Medicine, Uppsala University Hospital, Uppsala, Sweden

**Keywords:** PDGFRβ, Platelet-derived growth factor receptor beta, Pulmonary fibrosis, Pericytes, Positron emitted tomography, Molecular imaging, Fibrogenesis

## Abstract

**Background:**

Lung diseases such as idiopathic pulmonary fibrosis and acute respiratory distress syndrome (ARDS) are associated with significant morbidity and mortality, with limited treatment options. Platelet-derived growth factor receptor beta (PDGFRβ) signaling pathway is a key driver of fibrogenesis in different organs. In the lungs, pericytes have a high PDGFRβ expression, and their role as immune regulators and progenitors of myofibroblasts is increasingly recognized. Non-invasive techniques to assess active lung tissue remodeling are needed to improve disease monitoring and treatment evaluation. This study aimed to evaluate [^18^F]TZ-Z09591, targeting PDGFRβ, for imaging pulmonary injuries in human biopsies, and in vivo in animal models of lung injury.

**Results:**

[^18^F]TZ-Z09591 demonstrated high and specific binding to PDGFRβ-expressing cells. Autoradiography confirmed tracer uptake in lung injuries, including fibrotic foci, from human, rat, and pig lung tissues. In vivo positron emission tomography (PET) imaging of bleomycin-induced lung fibrosis in rats and an ARDS pig model showed significantly increased uptake in diseased lung segments compared to controls, especially in pulmonary injuries with collagen deposition, despite moderate background uptake.

**Conclusions:**

This study demonstrated that [^18^F]TZ-Z09591 can assess PDGFRβ expression in pulmonary injuries, supporting its potential for non-invasive assessment of lung tissue remodeling. PET imaging targeting PDGFRβ could improve disease monitoring, and provide new insights into pulmonary fibrosis progression.

**Supplementary Information:**

The online version contains supplementary material available at 10.1186/s41181-025-00366-3.

## Background

Acute and chronic lung diseases, including idiopathic pulmonary fibrosis (IPF) and acute respiratory distress syndrome (ARDS), pose significant clinical challenges due to their high morbidity, mortality, and limited treatment options. IPF, the most common fibrosing interstitial lung disease, is characterized by progressive fibrosis and poor prognosis (Sesé et al. 2020), while ARDS is an acute inflammatory lung injury associated with high mortality rates (30–50%) (Wick et al. 2024). These conditions ultimately lead to respiratory failure, highlighting the urgent need for better biomarkers to assess disease activity and guide therapeutic strategies.

Platelet-derived growth factor receptor beta (PDGFRβ) is a receptor tyrosine kinase expressed on pericytes and myofibroblasts (Bonner 2004; Henderson et al. 2013) which are key mediators of tissue remodeling and fibrosis. PDGFRβ signaling promotes fibroblast proliferation and pericyte-to-myofibroblast transition (Wang et al. 2019; Sun et al. 2019), contributing to extracellular matrix deposition and scar formation. Studies in rodent models have demonstrated that inhibiting PDGFRβ signaling attenuates fibrosis, underscoring its role as a pro-fibrotic mediator (KiShi et al. 2019). In addition, Nintedanib, one of the treatments currently approved for IPF, acts as an inhibitor of PDGFR α and β signalling as part of its mechanism of action (Wollin et al. 2015). Given its involvement in fibrogenesis, PDGFRβ is emerging as a promising biomarker of disease activity in lung fibrosis and repair.

Assessing PDGFRβ expression in the lung could provide valuable insights into fibrogenesis which traditionally requires invasive biopsies. Non-invasive imaging techniques such as positron emission tomography (PET) offer a promising alternative, allowing assessment of fibrosis progression. PET imaging with radiotracers targeting fibroblast activation protein (FAP) has demonstrated strong uptake in fibrotic lung regions (Bergmann et al. 2021; Röhrich, et al. 2022; Mori et al. 2024; Rosenkrans et al. 2022). PDGFRβ imaging could provide complementary insights by targeting another fibrogenic signaling pathway and notably pericytes, which are increasingly recognized as key contributors to fibrogenesis (Garrison et al. 2023; He et al. 2024; Wilson et al. 2018).

Our previous studies have validated PDGFRβ-targeted PET using the radiolabeled Affibody molecule Z09591 (also known in the literature as ATH001) for detecting fibrogenic cells in liver fibrosis models, suggesting its potential for translation to pulmonary fibrosis (PF) and other fibrotic diseases (Wegrzyniak et al. 2023, 2024). A gallium-68-labeled version of Z09591 ([^68^Ga]Ga-DOTA-Cys-ATH001) is currently in clinical development for the detection of liver fibrogenesis (NCT06562361) (Antaros Medical 2024). Here, we aimed to investigate the biodistribution and PDGFRβ-targeting efficacy of [^18^F]TZ-Z09591, evaluating its potential as a PET tracer for pulmonary injury assessment.

## Results

### ***Synthesis of [***^***18***^***F]TZ-Z09591***

Starting from 8 to 15 GBq of [^18^F]fluoride, the isolated radiochemical yield of [^18^F]MeTz was 18 ± 4%, and the radioactivity yield of [^18^F]TZ-Z09591 was 312 ± 34 MBq (n = 5). The radiochemical purity exceeded 99%. The molar activity at the end of synthesis was 18 MBq/nmol, estimated by dividing the molar amount of affibody molecule used in the labelling reaction by the activity of the purified and formulated [^18^F]TZ-Z09591 product solution.

### [^18^F]TZ-Z09591 binding to human lung tissue

**Fig. 1:**
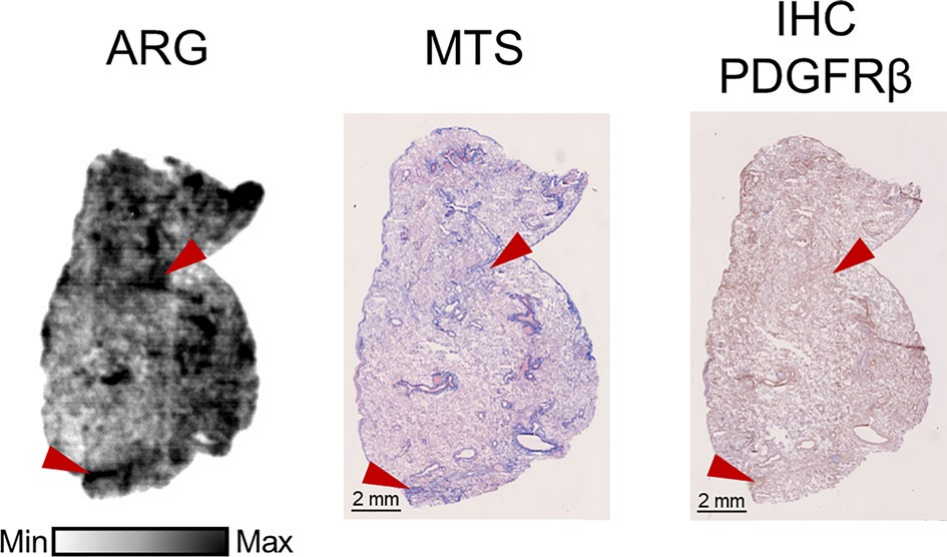
[^**18**^F]TZ-Z09591 binding in human lung lesions. Representative images of in vitro autoradiography (ARG), Masson's trichrome staining (MTS), and immunohistochemistry (IHC) targeting PDGFRβ in a lung biopsy from a patient with pulmonary fibrosis. Red arrows indicate fibrotic areas

PDGFRβ is expressed in the normal parenchyma of human lungs, but with a visibly stronger and more concentrated expression in areas of collagen deposition, corresponding to fibrotic lesions. Similarly, the autoradiography (ARG) performed on human biopsies revealed a generally homogeneous, moderate binding in normal parenchyma, with stronger binding colocalizing with lung fibrotic foci (Fig. 1). In addition, both the homogeneous background binding and the lesion-specific binding of [^18^F]TZ-Z09591 were blocked when the tissue was pre-incubated with Z09591-cys in high concentration, demonstrating the specificity of the binding observed in non-blocking conditions (Fig.S3). Single cell RNA sequencing (scRNAseq) of human pulmonary cells revealed that *PDGFRβ* is predominantly expressed in pericytes, vascular smooth cells, fibroblasts and myofibroblasts (Fig. S1).

### In vivo [18F]TZ-Z09591 uptake in xenograft model

**Fig. 2:**
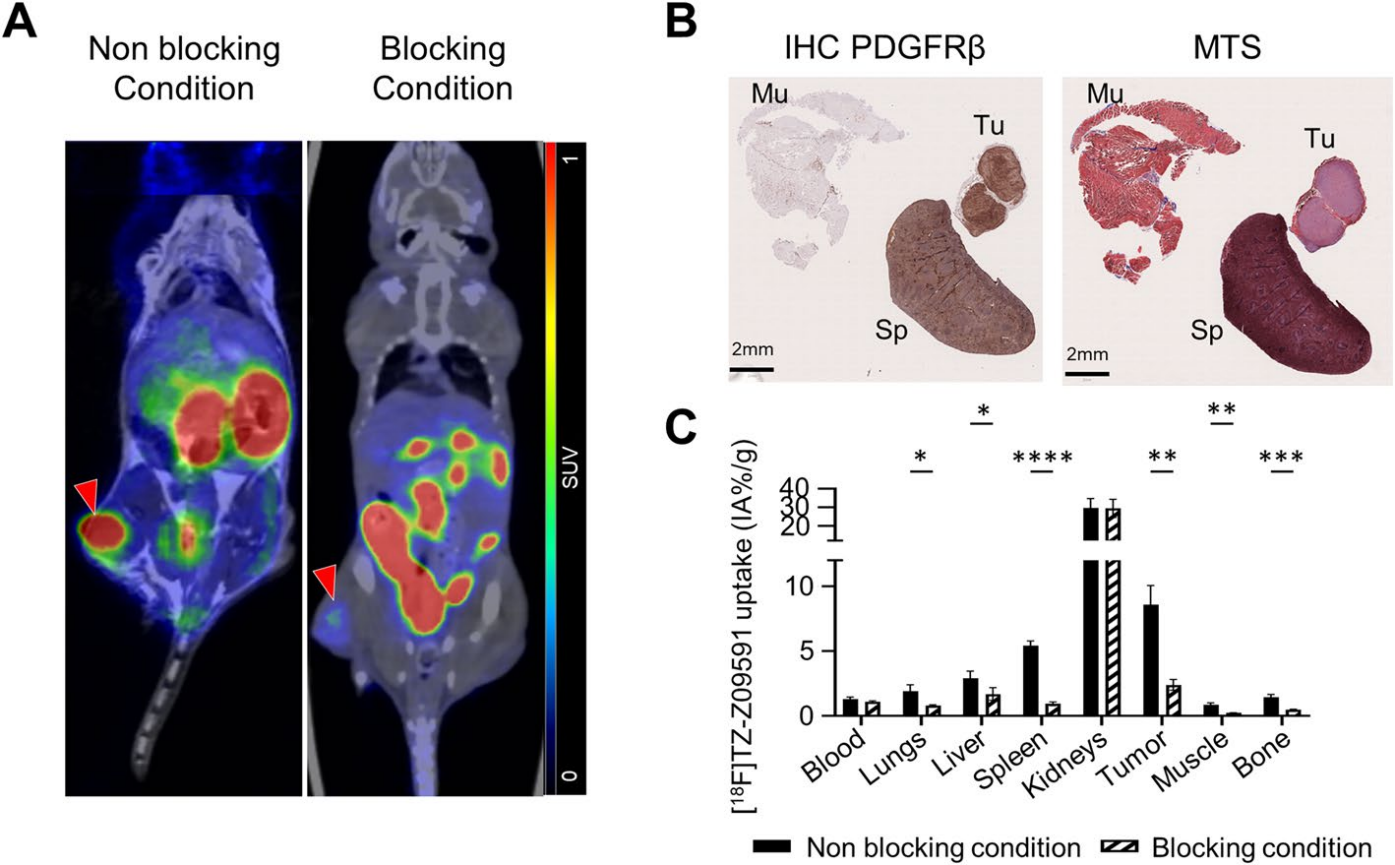
[^18^F]TZ-Z09591 uptake in the U-87 xenograft mouse model. **A** Representative PET/CT images of mice bearing U-87 tumors in the left hind limb (red arrows) under non-blocking and blocking conditions. **B** Representative immunohistochemistry (IHC) staining for PDGFRβ and Masson’s trichrome staining (MTS) in muscle (Mu), spleen (Sp), and tumor (Tu) biopsies from xenografted mice. **C** Tracer uptake (IA%/g) from ex vivo biodistribution analysis comparing blocking (n = 4) and non-blocking conditions (n = 4). Data are represented as mean +/− SD. ****p < 0.0001, ***p < 0.001, **p < 0.01, *p < 0.05. CD, caudal; CR; cranial; LE, Mu, muscle; left; Sp, spleen; RT, right; SUV, standardized uptake value; Tu, tumor

The expression of PDGFRβ in U-87 tumor xenografts was confirmed by immunohistochemistry (IHC), which also revealed PDGFRβ expression in the spleen (Fig. 2B). PET imaging and ex vivo biodistribution analyses demonstrated high tracer uptake in PDGFRβ-expressing tumors one hour post-injection (Fig. 2A, C). Blocking studies confirmed uptake specificity, as pre-treatment with a high concentration of Z09591-cys significantly reduced tumor uptake (8.58 ± 1.48%IA/g vs. 2.40 ± 0.42%IA/g, *P* = 0.001) (Fig. 2A, C). A similar strong blocking effect was observed in the spleen, where uptake decreased from 5.42 ± 0.35%IA/g to 0.93 ± 0.15%IA/g (*P* < 0.0001).

Among all organs, the spleen and tumor showed the most pronounced uptake reductions under the blocking condition (Δ = 4.49%IA/g and Δ = 6.18%IA/g, respectively). However, a significant decrease was also observed in the lungs (*P* = 0.0017, Δ = 1.14%IA/g), likely due to PDGFRβ expression in pericytes and vascular smooth muscle cells (VSMCs) (Fig. S2).

Muscle, bone and liver also exhibited minor reductions in uptake from low baseline levels (muscle: *P* = 0.0017, Δ = 0.62%IA/g; bone: *P* = 0.00067, Δ = 0.99%IA/g; liver: *P* = 0.046, Δ = 1.22%IA/g).

### Assessment of [18F]TZ-Z09591 in a bleomycin-induced pulmonary fibrosis rat model

**Fig. 3:**
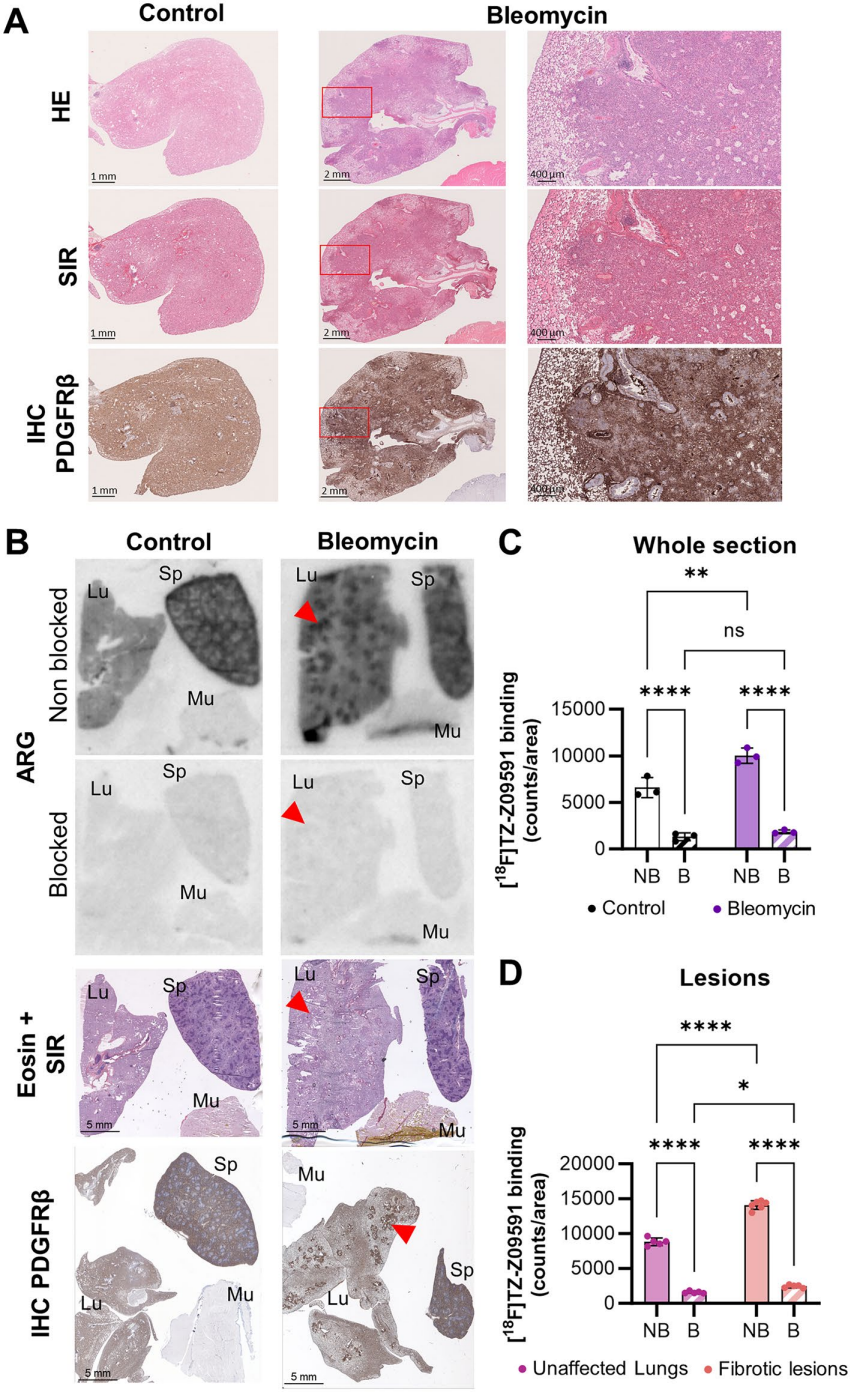
[^18^F]TZ-Z09591 in vitro binding in bleomycin-induced lesions in rats. Representative in vitro ARG images of lung (Lu), muscle (Mu), and spleen (Sp) biopsies from healthy control (**A**) and BLM-treated (**B**) rats under non-blocking and blocking conditions, alongside eosin-SIR staining of the same biopsies and PDGFRβ immunohistochemistry (IHC) from different biopsies of the same organs in the same rats. Quantification of [^18^F]TZ-Z09591 binding in whole lung sections (n = 3/condition) (**C**) and within pulmonary lesions (n = 5–6/condition) (**D**). ****p < 0.0001, **p < 0.01, *p < 0.05

One week after single intratracheal bleomycin (BLM) instillation, rats developed lung lesions characterized by collagen deposition and increased PDGFRβ expression (Fig. 3A, B). The severity of the BLM-induced lesions varied between animals (Figs. S5 and S6). In healthy murine lung parenchyma, PDGFRβ is moderately expressed, notably due to *Pdgfrβ* expression in pericytes (Fig. S2).

ARG revealed significantly higher [^18^F]TZ-Z09591 binding in lung sections from BLM-treated rats compared to healthy controls (*P* = 0.002) (Fig. 3B, C). Within the diseased lungs, tracer binding was significantly higher in lesions than in normal parenchyma (*P* < 0.0001) (Fig. 3B, D). Furthermore, pre-incubation with Z09591-cys effectively blocked tracer binding in lung sections, confirming the specificity of [^18^F]TZ-Z09591 for PDGFRβ-expressing regions.

**Fig. 4:**
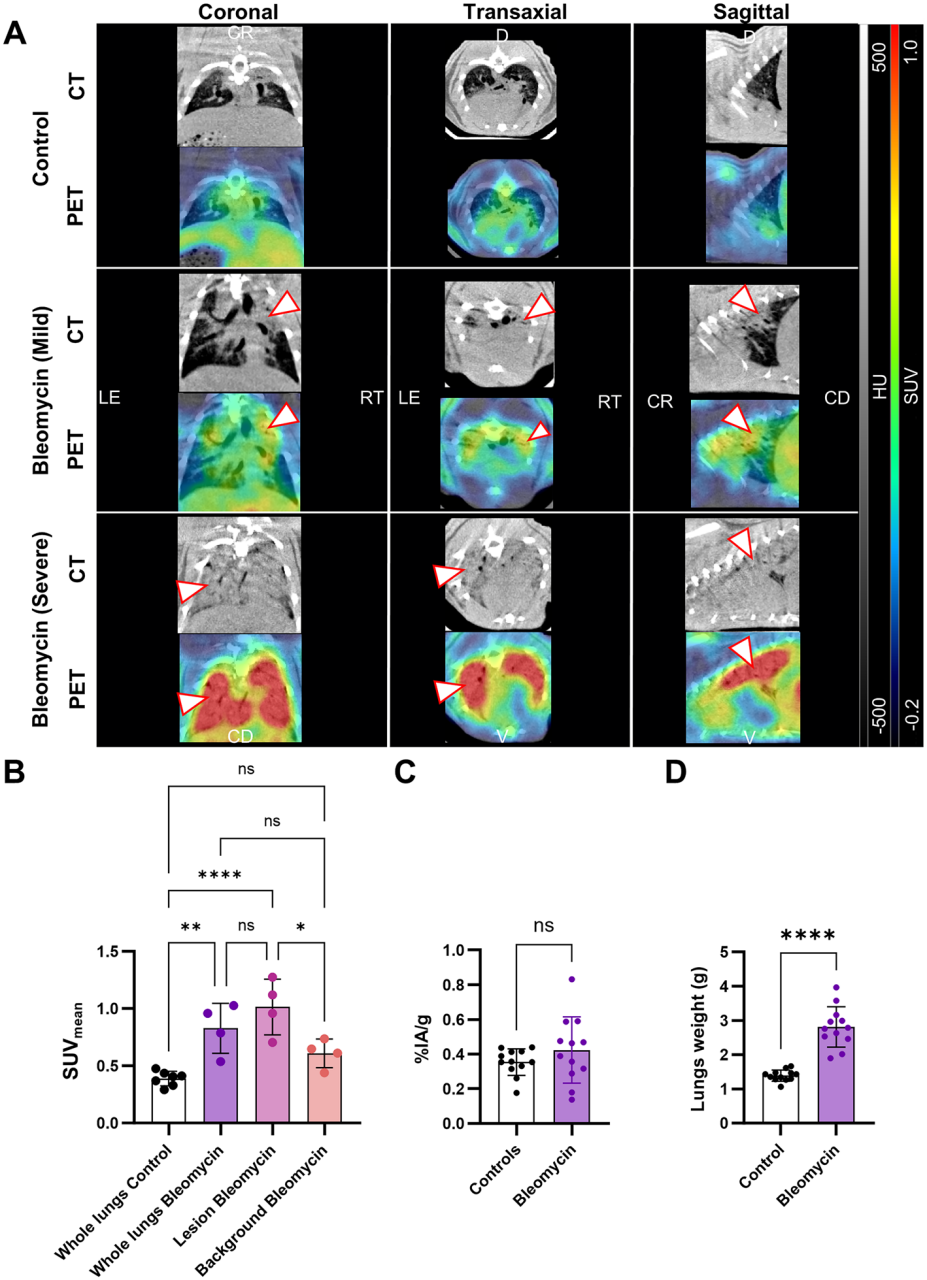
[^18^F]TZ-Z09591 post-mortem and ex vivo uptake in bleomycin-injured rat lungs. **A** Representative post-mortem PET/CT images of control and BLM-treated rats (mild and severe lung injuries), with [^18^F]TZ-Z09591 uptake in the lungs indicated by white arrows. **B** Quantification of [^18^F]TZ-Z09591 uptake in control (n = 7) and BLM-treated rats (n = 4), obtained from post-mortem PET/CT biodistribution analysis, and **C** ex vivo biodistribution (n = 12/group). **D** Comparison of lung weight between healthy lungs (n = 12) and BLM-injured (n = 12). ****p < 0.0001, **p < 0.01, *p < 0.05

In vivo PET imaging performed one hour post-injection revealed a significantly higher tracer uptake in the lungs of BLM-treated rats (mean standardized uptake value (SUV_mean_) = 0.827 ± 0.218) compared to healthy controls (SUV_mean_ = 0.389 ± 0.063) (*P* = 0.003), particularly in fibrotic lesions (SUV_mean_ = 1.014 ± 0.244) (Fig. 4A, B). The ex vivo ARG also revealed tracer binding localized to lesions within the lungs (Fig. S4). However, ex vivo biodistribution measurements of the whole lungs at the same time point did not show a significant difference in tracer uptake between the two groups (Fig. 4C). This discrepancy highlights the importance of analyzing specific lesions rather than whole-lung measurements, as signal from lung injuries may be diluted by surrounding normal tissue. The lungs of BLM-treated rats were significantly denser than those of healthy controls (2.811 ± 0.588 g and 1.390 ± 0.163 g respectively. *P* < 0.0001), likely due to increased inflammatory and fibrotic tissue (Fig. 4D).

In addition, stability studies in vitro and in vivo in rat plasma confirmed that the radiolabeled tracer remains stable over time (Fig. S7).

### [18F]TZ-Z09591 assessment in an ARDS Pig model

**Fig. 5:**
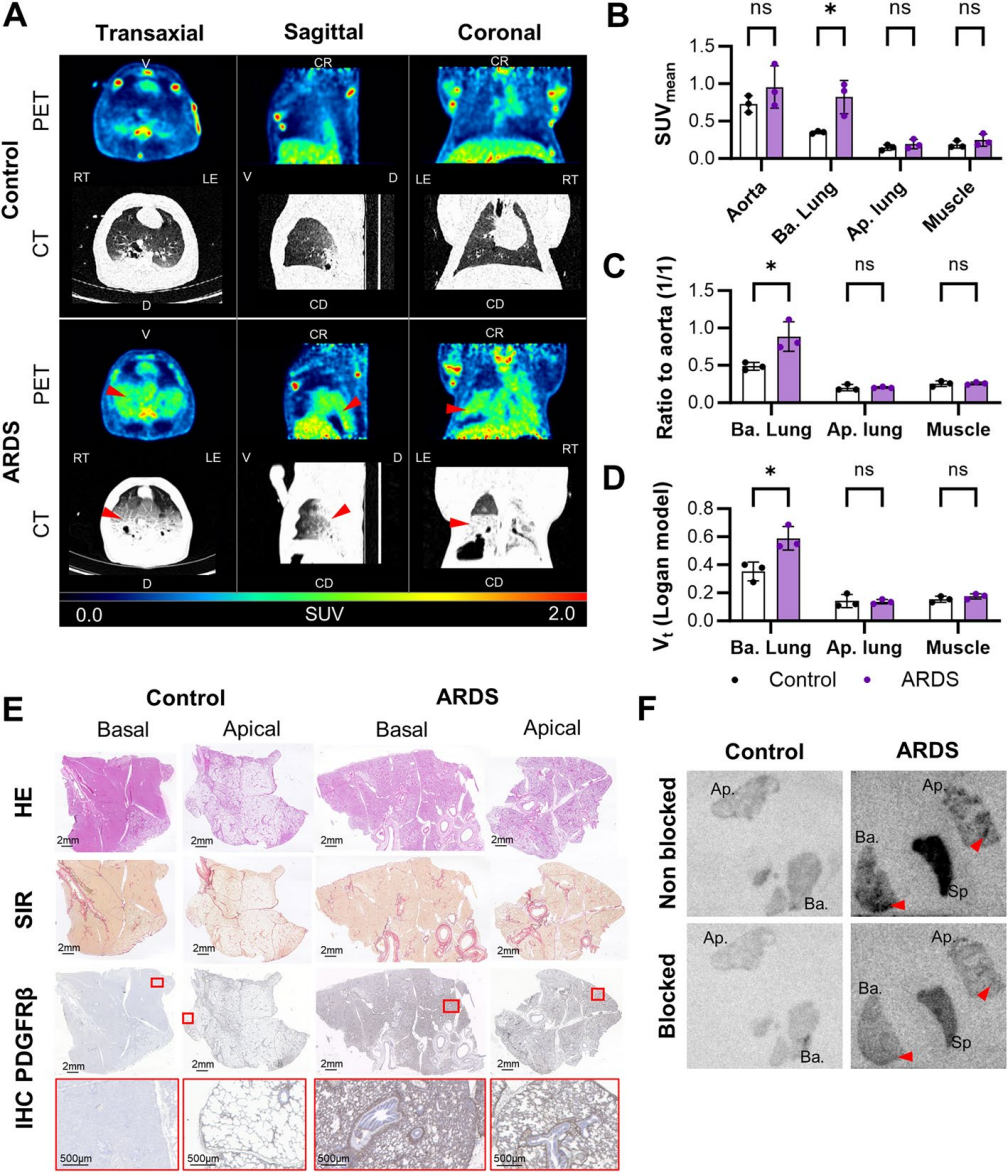
[^18^F]TZ-Z09591 uptake in pulmonary injury in an ARDS pig model. **A** Representative PET/CT images of a healthy pig and an ARDS pig. Red arrows indicate the basal lung regions in ARDS pigs with increased tracer accumulation. **B** Mean standardized uptake value (SUV_mean_) measurements from PET/CT imaging, comparing tracer uptake in the aorta, basal lung, and muscle between healthy (n = 3) and ARDS pigs (n = 3) at 60 min post-injection. **C** Tracer uptake ratios (relative to the aorta, at 60 min post-injection) and **D** Total distribution volume (Vt) in the basal lung, apical lung, and muscle. **E** Histological and immunohistochemical (IHC) staining of lung biopsies from the basal and apical regions, including Hematoxylin & Eosin (HE), Sirius Red (SIR), and PDGFRβ staining. **F** Representative in vitro autoradiography (ARG) image**s** in non-blocked and blocked conditions, showing tracer binding in basal lung, apical lung, and spleen biopsies from healthy and ARDS pigs. *p < 0.05. Ap., apical lung; Ba., basal lung; CD, caudal; CR; cranial; LE, left; RT, right; Sp, spleen; SUV, standardized uptake value; Vt, Total distribution volume

The lung function was significantly reduced in pigs with induced ARDS at the time of the scan (p/f = 78.0) compared to baseline (p/f = 546.0, *P* < 0.0001) and healthy control lungs (p/f = 466.7, *P* < 0.0001) (Fig. S8A). PET images of pigs injected with [^18^F]TZ-Z09591 showed significantly higher tracer uptake in the basal regions of ARDS lungs, where computed tomography (CT) abnormalities were also observed, compared to healthy controls (SUV_mean_ = 0.822 and 0.351 respectively, *P* value = 0.021) (Fig. 5A–C, Fig. S8B). However, perfusion was also increased in the basal regions of ARDS pigs (65.4 mL/min/100 g) compared to controls (26.4 mL/min/100 g) (Fig. S8C). Despite this, kinetic modeling correcting for blood radioactivity concentration and tracer delivery using total distribution volume (Vt) still revealed a significantly higher tracer uptake in the basal lungs of ARDS pigs compared to healthy pigs (Vt = 0.589 and 0.352, respectively. *P* value = 0.019). Additionally, in vitro ARG confirmed that the tracer binds more strongly to regions corresponding to lesions, as indicated by histology, in both basal and apical lung samples from ARDS pigs, while only low binding was observed in healthy control lungs and in unaffected lung parenchyma of ARDS animals. This binding was effectively blocked, demonstrating specificity (Fig. 5E, F).

In addition, stability studies in vitro and in vivo in pig plasma confirmed that the radiolabeled tracer remains stable over time (Fig. S7).

## Discussion

This study demonstrates the specific uptake of [^18^F]TZ-Z09591 in PDGFRβ-expressing tissues, particularly in pulmonary lesions, across two lung disease models: BLM-induced lung injuries in rats and ARDS in pigs.

PDGFRβ is a common marker of myofibroblasts, the primary extracellular matrix (ECM) producing cells in fibrosis in various organs, including the lungs (Bonner 2004; Henderson et al. 2013). ScRNA-seq studies (T.S. Adams et al., Fig. S1) have shown that PDGFRβ is expressed in lung fibroblasts, myofibroblasts, VSMCs, and pericytes, with particularly high expression in pericytes (Adams et al. 2020; He et al. 2018) (Figs. S1 and S2).

Pericytes are increasingly recognized as key contributors to pulmonary fibrogenesis (Garrison et al. 2023; He et al. 2024; Wilson et al. 2018). Their numbers were demonstrated to be elevated in diseased human lungs, including those of systemic sclerosis (SSc) patients (Valenzi et al. 2019). While their exact role remains incompletely understood, recent studies suggest that pericytes may function as immune regulators (Wilson et al. 2018; Barron et al. 2016; Hung et al. 2022) and, more importantly, as progenitors of myofibroblasts (Sun et al. 2019; Barron et al. 2016; Hung 2020; Hannan et al. 2021; Hung et al. 2013; Yamaguchi et al. 2020). PDGFRβ is a well-established biomarker of pericytes, and its signaling pathway is directly involved in pericyte epithelial-mesenchymal transition (EMT) (Wang et al. 2019; Sun et al. 2019), enabling their critical contribution to the myofibroblast pool. Previous studies have shown that inhibiting PDGFRβ signaling can prevent fibrosis progression and even reverse fibrosis in later stages in BLM-treated rats (KiShi et al. 2019). These findings highlight the potential of PDGFRβ as both a biomarker for fibrogenesis and a therapeutic target in PF.

Our findings align with prior studies demonstrating high PDGFRβ expression in pulmonary fibrotic foci (Henderson et al. 2013; KiShi et al. 2019; Yamaguchi et al. 2020; Ogawa et al. 2008). In vitro ARG using [^18^F]TZ-Z09591 revealed high and specific binding to fibrotic foci in human, rat, and pig lung sections (Figs. 1, 3, 4). Additionally, ARG showed moderate, homogeneous background binding in lung sections, which was blockable, confirming specificity. This background signal likely reflects PDGFRβ expression in pericytes and VSMCs beyond fibrotic lesions.

Before assessing [^18^F]TZ-Z09591 in lung disease models, we validated its in vivo specificity in mice xenografted with PDGFRβ-expressing U-87 cells. PET imaging and ex vivo biodistribution confirmed high tumor tracer uptake, which was significantly reduced by pre-treatment with Z09591-cys, demonstrating target specificity. These findings are consistent with previous studies using Z09591-based radiotracers in U-87 xenografted mice (Wegrzyniak et al. 2024; TolmacHev et al. 2014; Strand et al. 2014). Interestingly, the lungs exhibited moderate uptake, which was also blockable, confirming the presence of a moderate background of specific uptake in healthy murine lung tissue.

Additionally, we have demonstrated that [^18^F]TZ-Z09591 in vivo uptake was significantly higher in diseased lungs, especially in injuries. Previously, we demonstrated similar high, specific uptake in hepatic fibrotic foci in a liver fibrosis model (Wegrzyniak et al. 2023, 2024). However, ex vivo whole lung biodistribution in the BLM model did not show significant differences between treated and control lungs. This discrepancy may be due to local heterogeneity in fibrosis severity among BLM-treated rats and the presence of baseline PDGFRβ expression in healthy lungs, complicating early fibrosis detection when measuring whole-lung uptake. Unlike ex vivo biodistribution, PET imaging enables lesion-specific identification and analysis, where [^18^F]TZ-Z09591 demonstrated a 50–150% increase of in vivo binding in both rat and pig lung, compared to healthy lung areas.

Despite promising results, our study has some limitations. The BLM-treated rats were sacrificed at one week after a single intra-tracheal BLM instillation. This timepoint was chosen as it corresponds to the transition from the inflammatory phase to the early fibrogenic phase (Liu et al. 2017), a critical window for which reliable, non-invasive assessment methods are currently lacking. The use of the day 7 post-BLM model is supported by literature demonstrating upregulation of PDGFRβ and activation of fibrogenic pathways at this stage, including increased expression of α-SMA, procollagen-1Aα, and fibronectin (Kadam and Schnitzer 2024). Histopathological analysis confirmed moderate to severe fibrotic lesions with collagen deposition in the lungs, validating the model’s relevance for evaluating fibrogenesis. However, inter-individual variability in disease progression led to differences in injury severity among animals, and asymmetry between the lungs introduced variability in whole-lung tracer uptake measurements. Additionally, only male rats and pigs were used, and potential sex-related differences in PDGFRβ expression or tracer uptake were not investigated.

PF is a chronic, progressive disease characterized by irreversible extracellular matrix accumulation, leading to impaired gas exchange and, ultimately, respiratory failure.

IPF, for instance, is associated with poor prognosis and limited therapeutic options (Khor 2020). Current diagnostic approaches rely on high-resolution CT, which detect established fibrotic scars but lack sensitivity for early-stage disease. This limitation can delay diagnosis and therapeutic intervention (Hoyer et al. 2022). Lung biopsy with histological investigation can provide insights into fibrotic activity but is invasive and subject to sampling variability. Blood-based biomarkers have emerged as non-invasive alternatives (White et al. 2016; Clynick et al. 2022), but they lack lung specificity and may be influenced by comorbidities.

In contrast, ARDS represents a severe, acute form of lung injury, as seen in severe COVID-19 cases. Survivors may develop long-term sequelae, including pulmonary fibrosis (Hama Amin et al. 2022; Burnham et al. 2013). Diagnosing ARDS and predicting fibrotic progression remain challenging, as diagnosis relies on clinical assessment and imaging modalities such as chest X-ray or CT, which lack sensitivity to detect early fibrotic changes (Bellani et al. 2016, 2020; Sjoding et al. 2018).

These challenges highlight the need for a non-invasive imaging tool capable of detecting active tissue remodeling. PET imaging targeting PDGFRβ, a key mediator of fibrogenesis, has the potential to fill this gap. Indeed, it could enable earlier diagnosis and risk stratification in IPF and ARDS, guide treatment decisions, and allow dynamic monitoring of disease progression.

Furthermore, the most immediate and impactful application of PDGFRβ PET imaging may lie in clinical trial design. Indeed, it potentially offers a means to dynamically assess treatment response and improve patient selection by identifying patients with ongoing fibrogenesis in specific tissues.

PET imaging of FAP is being explored using for instance, [⁶⁸Ga]Ga-FAPI-46, in both cancer (Loktev et al. 2018; Shi et al. 2021; Kömek et al. 2022; Pang et al. 2020) and fibrotic diseases (Bergmann et al. 2021; Röhrich, et al. 2022; Mori et al. 2024; Rosenkrans et al. 2022; Pirasteh et al. 2022; Song et al. 2024; Rao et al. 2024; Yang et al. 2023; Sviridenko et al. 2022). While FAP PET imaging shows promise for monitoring fibrosis progression, the role of FAP in lung fibrotic diseases remains unclear, with studies suggesting both protective and pro-fibrotic effects (Fan et al. 2016; Egger et al. 2017). In contrast, PDGFRβ is expressed in a broader range of fibrogenic cells, particularly pericytes, which are gaining attention as myofibroblast progenitors (Sun et al. 2019; Barron et al. 2016; Hung 2020; Hannan et al. 2021; Hung et al. 2013). Given its direct involvement in pericyte transition to myofibroblast, PDGFRβ-targeted imaging may provide complementary insights to FAP imaging in pulmonary fibrosis.

The Affibody molecule Z09591 has already demonstrated potential in assessing liver fibrogenesis (Wegrzyniak et al. 2023, 2024). A ^68^Ga-labeled version of the Affibody molecule, named [^68^Ga]Ga-DOTA-Cys-ATH001 has entered clinical phase 1 development as an imaging agent for detection of PDGFRβ in metabolic dysfunction-associated steatohepatitis (MASH) (NCT06562361)^18^, making its future validation in pulmonary fibrosis imaging a next step.

## Conclusions

This study demonstrates that [^18^F]TZ-Z09591 specifically targets PDGFRβ-expressing cells in pulmonary lesions, reinforcing its potential as a non-invasive PET imaging tracer for assessing tissue remodeling. Given the need for better diagnostic tools in pulmonary fibrosis, this tracer could aid in early detection, patient stratification, and treatment monitoring. With a gallium-68 labeled Affibody molecule Z09591 already in clinical evaluation for liver fibrosis, its translation to pulmonary applications is a promising next step.

## Methods

### Ethical considerations

Frozen lung biopsy sections from patients with pulmonary fibrosis (n = 3) were obtained from the Uppsala Biobank (#827). The use of human biopsies for ARG binding studies was approved by the Swedish Ethical Review Authority (approval number: 2024-00969).

All animal experiments were approved by the Animal Ethics Committee of the Swedish Animal Welfare Agency (approval numbers: 5.8.18-09018/2020, 5.8.18-08564/2019, and 5.8.18-15648/2019). All procedures were conducted in accordance with the ARRIVE (Animal Research: Reporting of In Vivo Experiments) guidelines and institutional policies, specifically the “Uppsala University Guidelines on Animal Experimentation” (UFV 2007/724).

### Synthesis of [18F]TZ-Z09591

The Z09591 Affibody molecule was synthesized by solid-phase peptide synthesis (Almac), functionalized with a single trans-cyclooctene (TCO) group at the C-terminus, and radiolabeled using a click chemistry approach by inverse Diels alder reaction tetrazine-TCO, following a previously described method (Wegrzyniak et al. 2023; Syvänen et al. 2020).

Briefly, cyclotron-produced [^18^F]fluoride was trapped on a Chromabond PS-HCO₃ Shorty/45 mg cartridge (Macherey–Nagel), washed with acetonitrile and eluted with solution of N,N,N-trimethyl-5-((2,3,5,6-tetrafluorophenoxy)carbonyl)pyridin-2-aminium chloride (10 mg) in acetonitrile (800 µL). During this elution step, the [^18^F]fluoride reacted instantly at room temperature, forming [^18^F]F-Py-TFP, which was subsequently passed over an Oasis MCX Short cartridge (Waters), pre-conditioned with acetonitrile (3 mL), to remove the precursor cation. The purified product was then transferred into a solution containing (4-(6-methyl-1,2,4,5-tetrazin-3-yl)phenyl)methanamine (1 mg) dissolved in DMSO (100 µL) and triethylamine (5 µL) in acetonitrile (100 µL). The mixture was reacted at 55 °C for 10 min, yielding the radiolabelled tetrazine intermediate [^18^F]MeTz. [^18^F]MeTz was purified by HPLC, formulated in ethanol by solid phase extraction using a tC18-SepPak (Waters). The ethanol was then removed under a stream of nitrogen gas and heating at 65 °C for 7 min before formulation in a PBS solution.

For conjugation, [^18^F]MeTz was incubated with the TCO-functionalized Z09591 Affibody molecule (100 µg, 14 nmol) in PBS (300 µL) at room temperature for 10–15 min. The final product, [^18^F]TZ-Z09591, was purified using a NAP-5 size exclusion column (Cytiva). Radiochemical purity was assessed by analytical HPLC, and identity was confirmed by co-injection with a TCO-conjugated PDGFRβ Affibody standard.

### Radiochemical purity assessment

The radiochemical purity was assessed by radio-HPLC (Agilent 1290 Infinity II system, Agilent Technologies, California, USA) using a Vydac 214 MS 5 µm C4 column (50 × 4.6 mm, Avantor, Pennsylvania, USA). The mobile phases consisted of A: 0.1% trifluoroacetic acid in water, and B: acetonitrile. A linear gradient from 5 to 80% B was applied over 10 min at a flow rate of 4 mL/min. The product eluted at a retention time of 4.3 min.

### In vitro experiments

#### Immunochemistry and staining

Biopsies from humans and animal models were fixed in 4% paraformaldehyde for 24 h, dehydrated in 70% ethanol, and embedded in paraffin. Sections (4 μm) were stained with hematoxylin–eosin (H&E), Sirius red (SIR), and Masson’s Trichrome (MTS) at Uppsala University Hospital using standard protocols. IHC staining for PDGFRβ was performed with the Autostainer Link 48 and EnVision FLEX High pH system (Agilent). Antigen retrieval was carried out using PT-Link (Dako) with High pH Target Retrieval Buffer The sections were then incubated with recombinant anti-PDGFRβ antibody (RRID: AB_777165, 1:300 dilution, 60 min), followed by HRP-conjugated secondary antibodies. Finally, stained sections were digitized with a NanoZoomer S60 (Hamamatsu) at 20× magnification and viewed using QuPath-0.2.3.

#### Autoradiography

For in vitro ARG, human and animal frozen tissue sections (20 µm) were pre-incubated in PBS with 1% bovine serum albumin (BSA) for 15 min at room temperature. In the blocking condition, 2 µM of unlabeled Z09591-cys was added to assess tracer specificity. After pre-incubation, [^18^F]TZ-Z09591 was added (5 nM, ~ 0.1 MBq/mL and incubated for 60 min. Unbound radiotracer was removed through sequential washes: twice in cold PBS/BSA (1%), once in PBS, and a final dip in MQ water. Sections were dried at 37 °C for 10 min. Calibration standards were prepared by spotting 10 µL of the incubation solution onto absorbent paper. Slides and standards were exposed to a phosphor-imaging plate (BAS-MS, FujiFilm) overnight and scanned using a phosphor imager (Amersham Typhoon FLA 9500, GE). Image analysis was performed with ImageJ (NIH, US).

For ex vivo ARG, biopsies were collected from mice injected with [^18^F]TZ-Z09591 and euthanized one hour post-injection. Tissues were snap-frozen, embedded in optimal cutting temperature compound (OCT), and cryosectioned at 20 µm using a Micron HM560 cryostat (Germany). Sections were mounted on Superfrost Plus slides (Menzel-Gläser). Slides and standards were exposed to a phosphor-imaging plate and scanned following the same protocol as described for in vitro ARG.

### Animal models

#### Housing and general care

Mice and rats (Taconic) were housed in groups of five and two per cage, respectively, with GLP Aspen Bedding (TAPVEI). They had ad libitum access to standard chow food and water. Housing conditions were maintained at 22 °C with 50% humidity under a 12-h light/dark cycle.

Pigs were acquired from a local breeder on the morning of the experiment.

### Xenograft tumor mouse model

#### U-87 tumor implantation

Xenografts of U-87 glioma cells were established in ten female BALB/C nu/nu mice (RRID: IMSR_TAC:BALBNU) through subcutaneous injection of 2 × 10^6^ U-87 cells per mouse (ATCC, RRID: CVCL_0022, Table S12) into the hind leg. Tumors were allowed to grow for 4 to 6 weeks before experimentation.

#### Ex vivo biodistribution

Eight xenografted mice received an intravenous injection of [^18^F]TZ-Z0959 with a target dose of 1 MBq (~ 0.3 µg peptide mass). To assess tracer specificity, four of these mice were pre-injected with 1 mg/kg Z09591-cys (blocking condition). One hour post-injection, the mice were euthanized, and the following organs were collected: blood, lungs, liver, spleen, kidneys, tumor, muscle, bone, and gastrointestinal tract. Each sample was weighed and measured for radioactivity using a gamma counter.

#### In vivo PET/CT imaging

Two xenografted mice underwent PET/ magnetic resonance (MR) imaging. They received [^18^F]TZ-Z09591 (10 MBq) via intravenous injection, with one mouse pre-injected with 1 mg/kg Z09591-cys (blocking condition). At 45 min post-injection, mice were anesthetized with sevoflurane and underwent a 30-min full-body static PET scan, followed by MR anatomical imaging.

### Bleomycin rat model of lung injury

#### Bleomycin administration

Lung injury was induced in twelve male Sprague Dawley rats (RRID: RGD_1566440) by a single intratracheal instillation of 1500 IU BLM in 200 µL of saline (2 mg/kg). The rats were monitored for one week post-injection before further experimentation. A control group of twelve healthy male Sprague Dawley rats was also included for comparison.

#### Ex vivo biodistribution

All rats received an intravenous injection of approximately 5 MBq [^18^F]TZ-Z09591 and were euthanized 1 h post-injection. Following euthanasia, the lungs, the muscle and the spleen were collected, weighed and their radioactivity measured by a gamma counter.

#### Post-mortem PET/CT imaging

Four of the BLM-treated rats, and seven controls underwent post-mortem PET/CT imaging. One hour post-injection, the rats were euthanized, and a 40 min static PET/CT scan was performed to assess tracer distribution in lung tissue.

### Pig model of lung injury and ARDS

#### ARDS induction

Six male Swedish Landrace pigs (~ 2 months old) were transported to Uppsala University, where all procedures were performed. Upon arrival, pigs were initially anesthetized with intramuscular tiletamine-zolazepam. They were then intubated, and sedation was maintained using intravenous ketamine, fentanyl, and midazolam.

Acute lung injury and ARDS were induced under full anesthesia in three pigs using a dual-hit approach starting with repeated lung lavages (35 mL/kg warm 0.9% NaCl) to remove lung surfactants, followed by injurious mechanical ventilation as previously described (Puuvuori et al. 2022). Ventilation was set to volume-controlled mode (fraction of inspiratory oxygen concentration (FiO_2_) = 0.5, positive End-Expiratory Pressure (PEEP) = 5 cmH_2_O, tidal volume = 6–8 mL/kg, respiratory rate = 20/min).

Up to eight lavages with 35 mL/kg warm 0.9% NaCl were administered, while lung function was continuously monitored by assessing oxygen saturation and the ratio of partial pressure of oxygen in arterial blood to the FiO_2_ (PaO_2_/FiO_2_, or p/f ratio). Once a p/f ratio below 100 mmHg (severe ARDS) was reached, injurious ventilation was started and maintained for 1 h (FiO_2_ = 1, PEEP = 0 cmH_2_O, respiratory rate = 30/min, peak pressure = 35 cmH_2_O).

Lung function and ARDS severity were monitored throughout maintenance of anesthesia until PET scanning.

Three pigs that did not undergo the dual-hit injury protocol served as controls.

#### In vivo PET/CT imaging

The three pigs with ARDS underwent [^18^F]TZ-Z09591 PET/CT imaging starting five to eight hours post-induction of lung injury, while healthy controls (n = 3) were scanned without a waiting period. A CT scan over the lungs was performed using a digital 4-ring, 64-slice CT scanner (GE Discovery MI) for attenuation correction. A 10-min dynamic PET scan was acquired over the lungs after intravenous administration of radiolabeled [^15^O]H_2_O to assess regional perfusion. Next, following an intravenous injection of [^18^F]TZ-Z09591 (target dose of 4 MBq/kg, ~ 3–5 µg peptide mass), a 60-min dynamic PET scan was performed over the lungs using a GE Discovery MI scanner (4 mm spatial resolution, 30 frames: 12 × 10 s, 6 × 30 s, 5 × 2 min, 5 × 5 min, 2 × 10 min). Plasma and whole blood radioactivity levels were measured using a gamma well counter at 5, 30, and 60 min post-injection to evaluate tracer pharmacokinetics and in vivo stability. PET images were reconstructed using the VPFX-S algorithm (GE Healthcare; OSEM, Time-of-Flight, Resolution Recovery: 3 iterations, 16 subsets, 3 mm post-filter, 256 × 256 matrix).

After PET imaging, a contrast enhanced CT scan was performed for anatomical reference. Lastly pigs were euthanized by intravenous KCl under deep anesthesia. Tissue biopsies were collected from the lungs (apical and basal regions), spleen, and liver, then processed for histological analysis (embedded in OCT and snap-frozen) and in vitro ARG to assess tracer binding at the cellular level.

#### PET/CT analysis

PET/CT images were analyzed using PMOD 4.0 (PMOD Technologies). For rat scans, tissue segmentation was performed on fused PET/CT images. SUV_mean_ values were extracted from a single PET frame for rat scans. For the pig scans, Regions of Interest (ROIs) were delineated on high resolution CT images, and SUV_mean_ for co-registered PET images were extracted for relevant tissues, including descending aorta and basal (clearly affected on CT images) and apical lung segments. The Vt for binding of [^18^F]TZ-Z09591 in lung tissue was estimated by the Logan graphical model using blood pool as input function (PKIN module, PMOD Technologies). The perfusion in each ROI was estimated from modeling of kinetic data from the [^15^O]H_2_O scan using the Water PET model with Flow and Dispersion (PKIN module, PMOD Technologies).

#### Statistical methods

Data are presented as mean ± standard deviation (SD). For Fig. 3C, D, 'n' refers to the number of lung sections and pulmonary lesions analyzed in ARG, respectively. In all other figures, ‘n’ represents the number of animals. Normality was assessed using the Shapiro–Wilk test, and variance equality was evaluated with an F-test. A significance threshold of 0.05 was applied (*p < 0.05, **p ≤ 0.01, ***p ≤ 0.001, ****p ≤ 0.0001). Statistical analyses were performed using GraphPad Prism 10.1 (GraphPad Software, San Diego, CA, USA). Detailed statistical information is provided in the supplementary data (Tables S1–S11).

## Supplementary Information

**Additional file 1.**

## Data Availability

The datasets used and/or analyzed during the current study are available from the corresponding author on reasonable request.

## Abbreviations

ARDS: Acute respiratory distress syndrome
ARG: Autoradiography
ARRIVE: Animal research: reporting of in vivo experiments
BLM: Bleomycin
BSA: Bovine serum albumin
ECM: Extracellular matrix
EMT: Epithelial-mesenchymal transition
FAP: Fibroblast activation protein
FiO_2_: Fraction of inspiratory oxygen concentration
H&E: Hematoxylin–eosin
IHC: Immunochemistry
IPF: Idiopathic pulmonary fibrosis
MASH: Metabolic dysfunction-associated steatohepatitis
MR: Magnetic resonance
MTS: Masson's trichrome staining
OA: Oleic acid
OCT: Optimal cutting temperature compound
PaO_2_: Partial pressure of oxygen in arterial blood
PDGFRβ: Platelet-derived growth factor receptor beta
PEEP: Positive end-expiratory pressure
PET: Positron emission tomography
PF: Pulmonary fibrosis
p/f: Ratio of partial pressure of oxygen in arterial blood to the fraction of inspiratory oxygen concentration (PaO_2_/FiO_2_)
ROI: Region of interest
SD: Standard deviation
SIR: Sirius red
SSc: Systemic sclerosis
SUV: Standardized uptake value
TCO: Trans-cyclooctene
TZ: Tetrazine
VSMC: Vascular smooth muscle cells
Vt: Total distribution volume
