# Supplementary material for: PET imaging of platelet derived growth factor receptor β in lung fibrosis
Source: EJNMMI Radiopharm Chem. 2025 Jul 15;10:44. doi: 10.1186/s41181-025-00366-3 (PMC12263540; doi:10.1186/s41181-025-00366-3)
Supplement: Supplementary file 1 — Additional file 1. [file 41181_2025_366_MOESM1_ESM.docx]

**PET imaging of Platelet Derived Growth Factor Receptor β in lung fibrosis**

Olivia Wegrzyniak et Al.

**Table of contents**

Supplementary methods…………………………………………………………………….…2

Figure S1………………………………………………………………………………………3

Figure S2………………………………………………………………………………………4

Figure S3………………………………………………………………………………………4

Figure S4………………………………………………………………………………………5

Figure S5………………………………………………………………………………………5

Figure S6………………………………………………………………………………………6

Figure S7………………………………………………………………………………………7

Figure S8………………………………………………………………………………………7

Table S1………………………………………………………………………………………..8

Table S2………………………………………………………………………………………..8

Table S3………………………………………………………………………………………..9

Table S4………………………………………………………………………………………..9

Table S5………………………………………………………………………………………10

Table S6………………………………………………………………………………………10

Table S7………………………………………………………………………………………10

Table S8………………………………………………………………………………………11

Table S9………………………………………………………………………………………11

Table S10..……………………………………………………………………………………11

Table S11..……………………………………………………………………………………12

Table S12..…………………………………………………………..……………………..…12

# **Supplementary methods**

## *Single cell expression analysis of open reference lung datasets*

The two-dimensional UMAP plots depicting human *PDGFRB* gene expression in epithelial, stromal, and immune cells in healthy and IPF lung tissue, as well as the violin plot illustrating *PDGFRB* expression across different cell types, were generated using data from T. S. Adams et al.^1^, as available in the Idiopathic Pulmonary Fibrosis Cell Atlas^2^. The bar chart showing the average expression of *Pdgfrβ* in lung cells from adult mice was extracted from the RNA-seq databases of Betsholtz Lab^3^, based on the work of He, L. et al.^4^

## *Plasma Stability Assessment*

In vivo stability of [¹⁸F]TZ-Z09591 was evaluated in plasma from arterial blood collected from rats and pigs at 0, 5, 30, and 60 minutes post-injection. Plasma was isolated by centrifugation (3000 × g, 2 min, 4°C), mixed 1:1 with acetonitrile for protein precipitation, incubated at 37°C (except at T₀), and centrifuged again (16,000 × g, 2 min, 4°C). Supernatants were filtered through a 0.2 μm nylon membrane and analyzed by radio-HPLC (VWR HITACHI system) using a Vydac C4 214MS reverse-phase column (50 × 4.6 mm, 5 μm) with a gradient elution of 0.1% TFA in water and acetonitrile. The intact tracer eluted at ~4.6 min; free [¹⁸F]fluoride at <1 min. Data acquisition and analysis were performed using Agilent OpenLAB Chromaster EZChrome Edition software.

In vitro stability was assessed by incubating 50 μL of [¹⁸F]TZ-Z09591 with 150 μL of phosphate-buffered saline (PBS), rat, or human plasma at 0, 5, 30, 60, 90, 120, 150, and 180 minutes. PBS samples were incubated at room temperature, while plasma samples were maintained at 38°C. Samples were analyzed by radio-TLC using silica RP-18 plates with 8.1 mM ammonium carbonate/acetonitrile (50:50) as the mobile phase.

Methods were adapted from previously described protocols^5,6^.


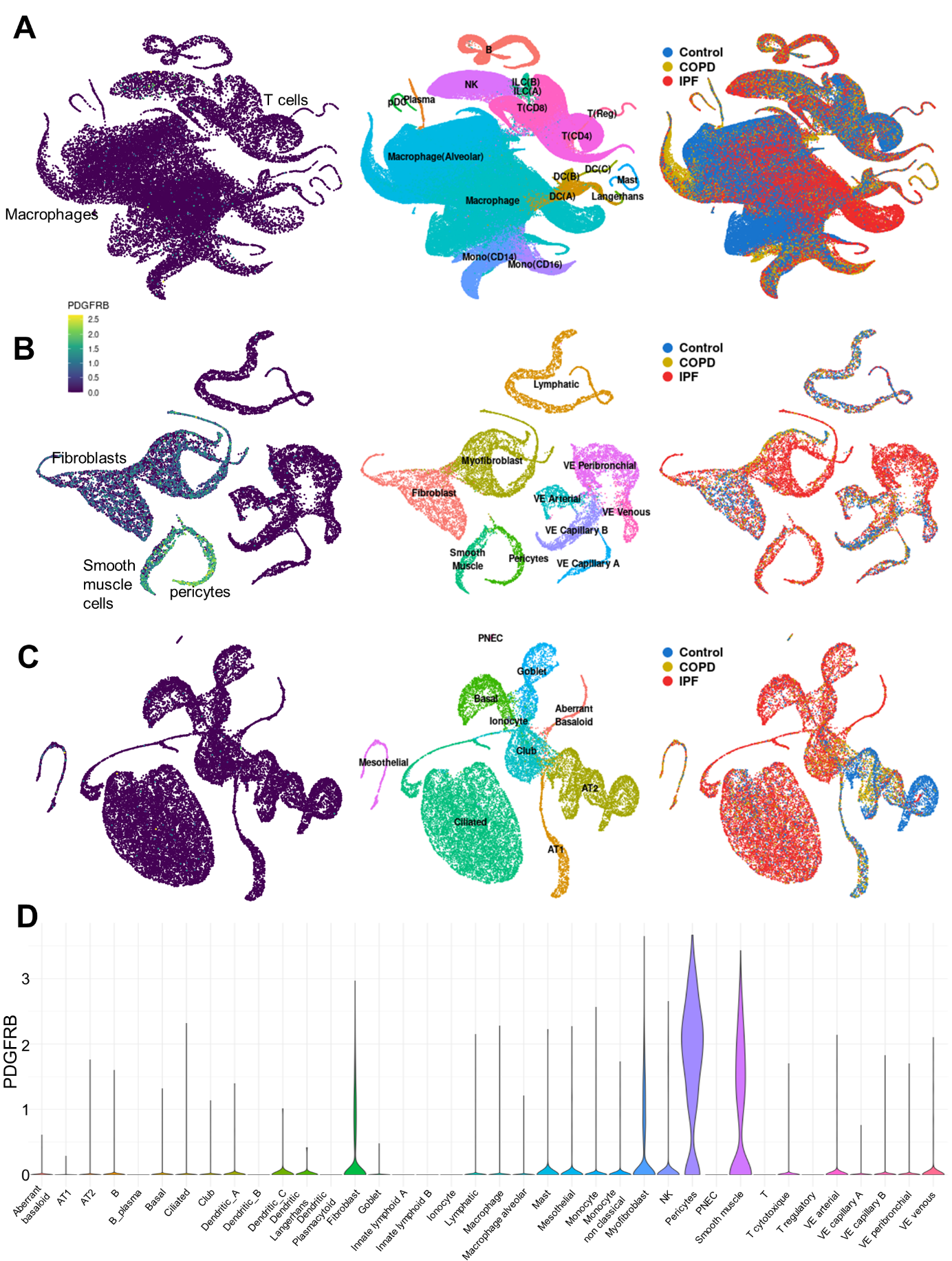


**Figure S1.** UMAPs of  immune cells (A), endothelial and mesenchymal cells (B), and epithelial cells (C) from IPF, COPD, and control lungs labeled by PDGFRB expression (left), cell type (middle), and disease status (right). (D) Violin plots showing PDGFRB expression across different cell types. Data extracted from the IPF atlas^1^.


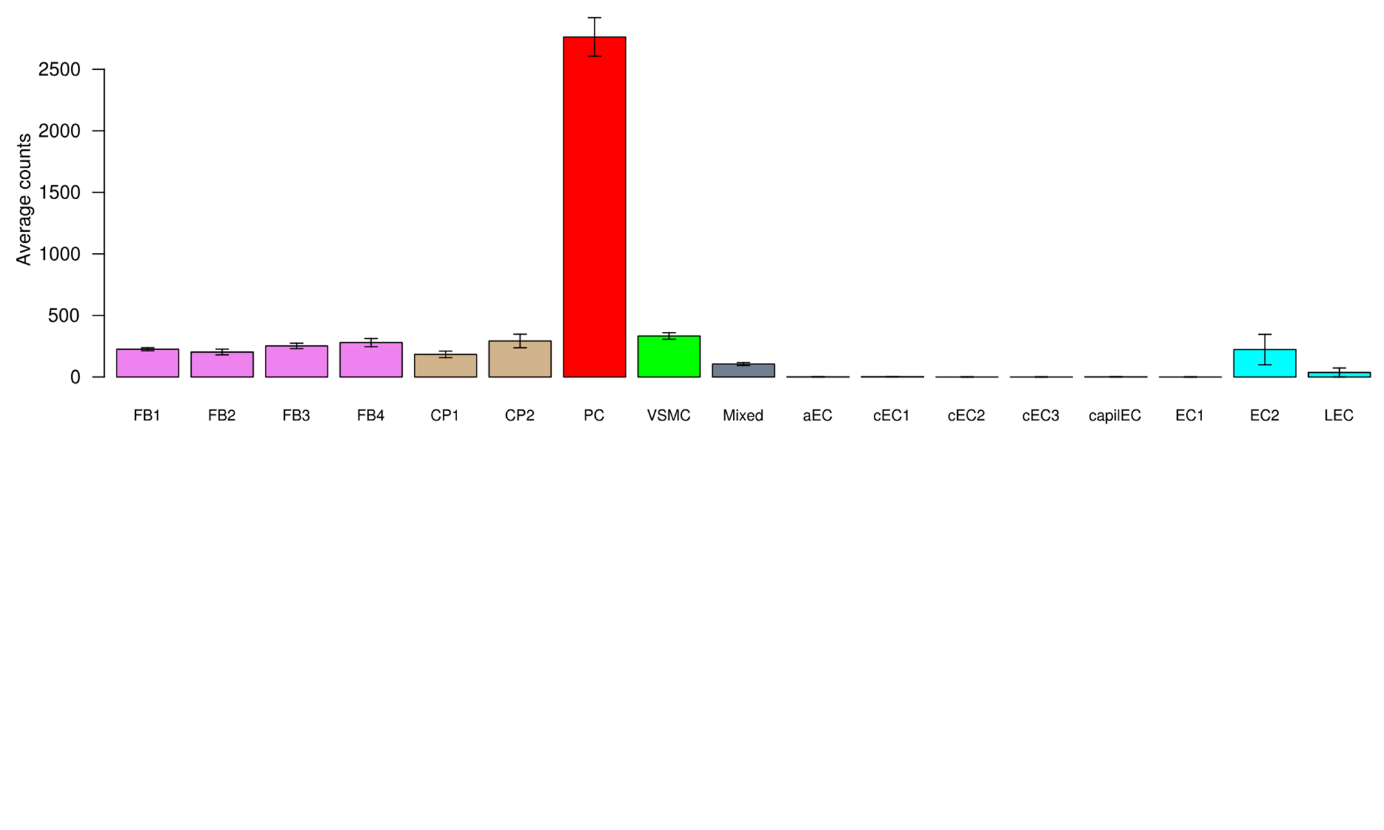


**Figure S2.** scRNAseq data showing average PDGFRB expression accross different cell clusters in adult mouse lung . Data sourced from the Betsholtzlab Single-cell RNAseq databases^4^. FB, Vascular fibroblast-like cells; CP, Cartilage perichondrium; PC, Pericytes; VSMC, Vascular smooth muscle cells; EC, Endothelial cells; capil, capillary; a, arterial; c, continuum; L, Lymphatic; 1,2,3,4, subtypes.


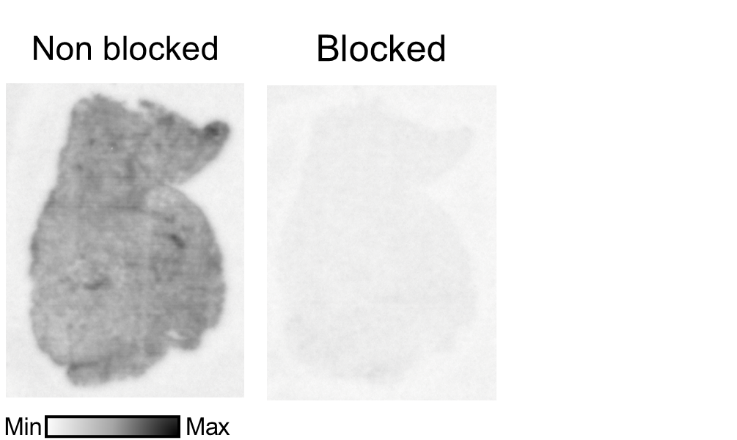


**Figure S3.** Representative images of in vitro ARG in a lung biopsy from a lung cancer patient with mild fibrosis, in non blocked and blocked condition.


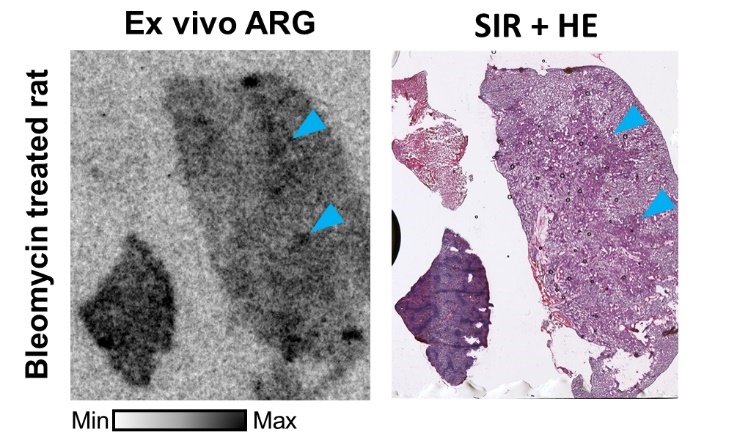


**Figure S4.** Ex vivo ARG images of lung (Lu), muscle (Mu), and spleen (Sp) biopsies from bleomycin-treated rats, alongside HE-SIR staining of the same biopsies. Blue arrows indicate lung lesions with tracer accumulation.


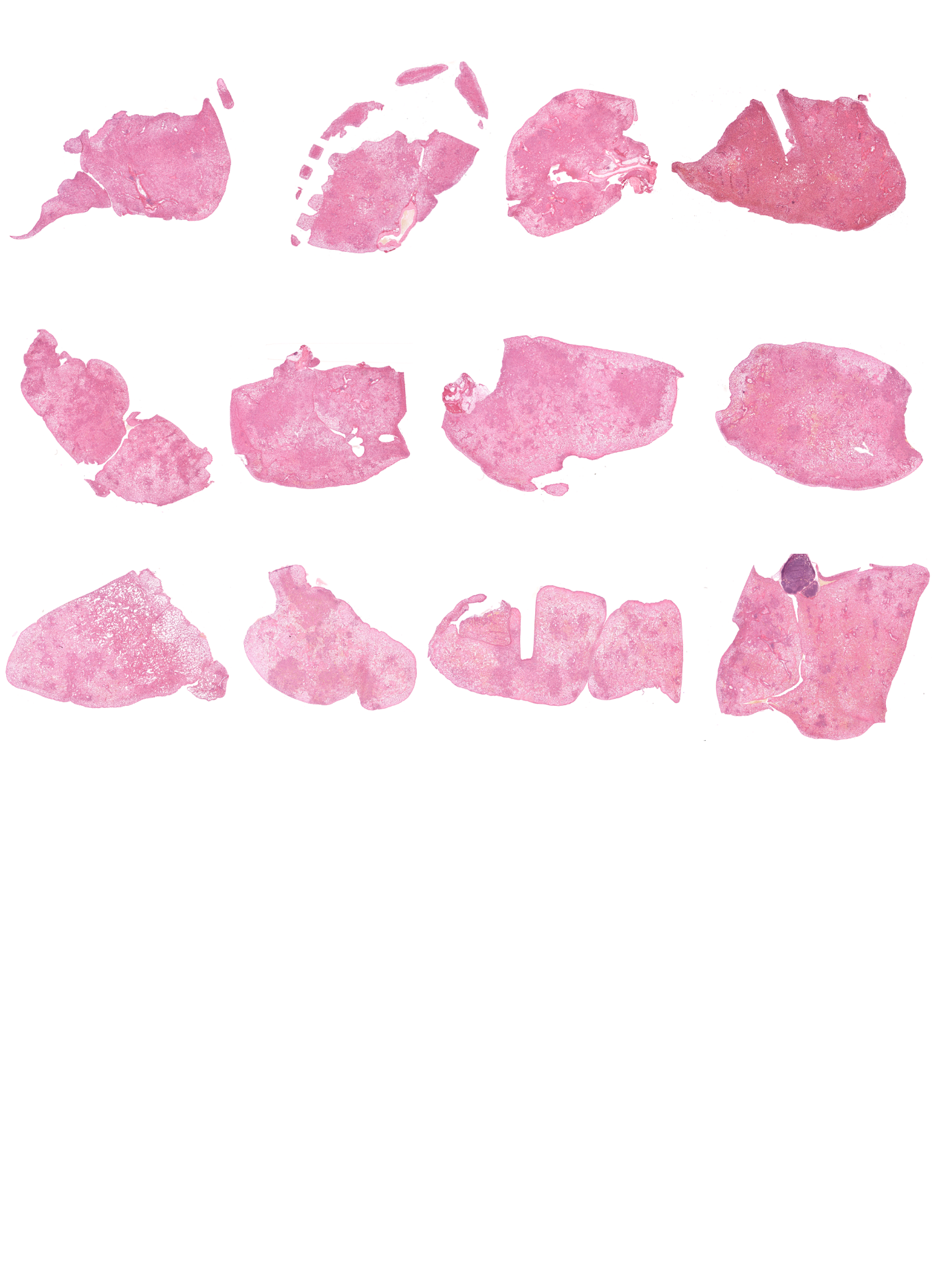


**Figure S5.** SIR staining of lung sections from all BLM-treated rats (n = 12), illustrating inter-individual variability in fibrosis severity.


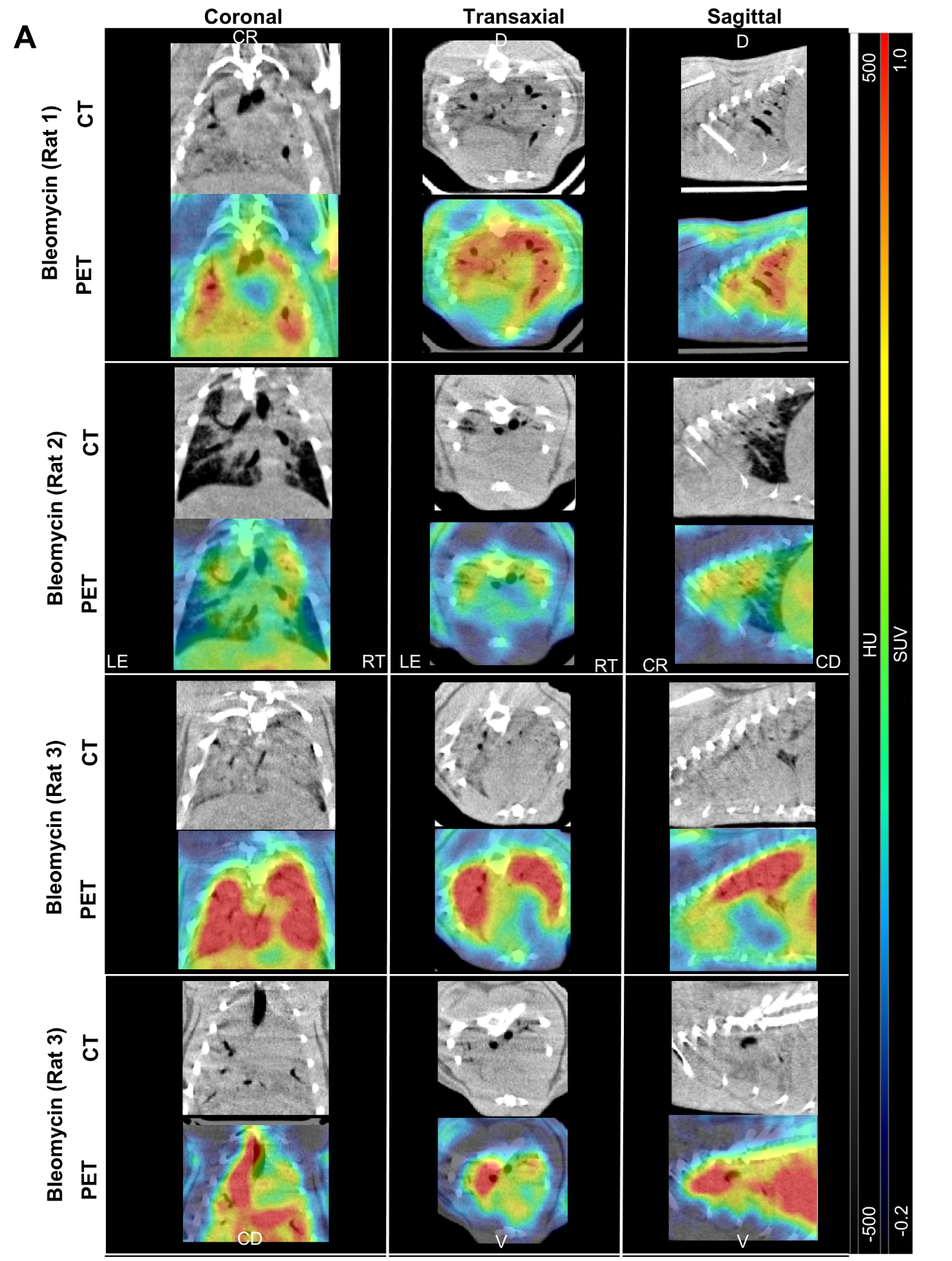


**Figure S6.** Representative PET images of all BLM-treated rats (n = 4) acquired post-mortem at 1 hour post tracer injection.


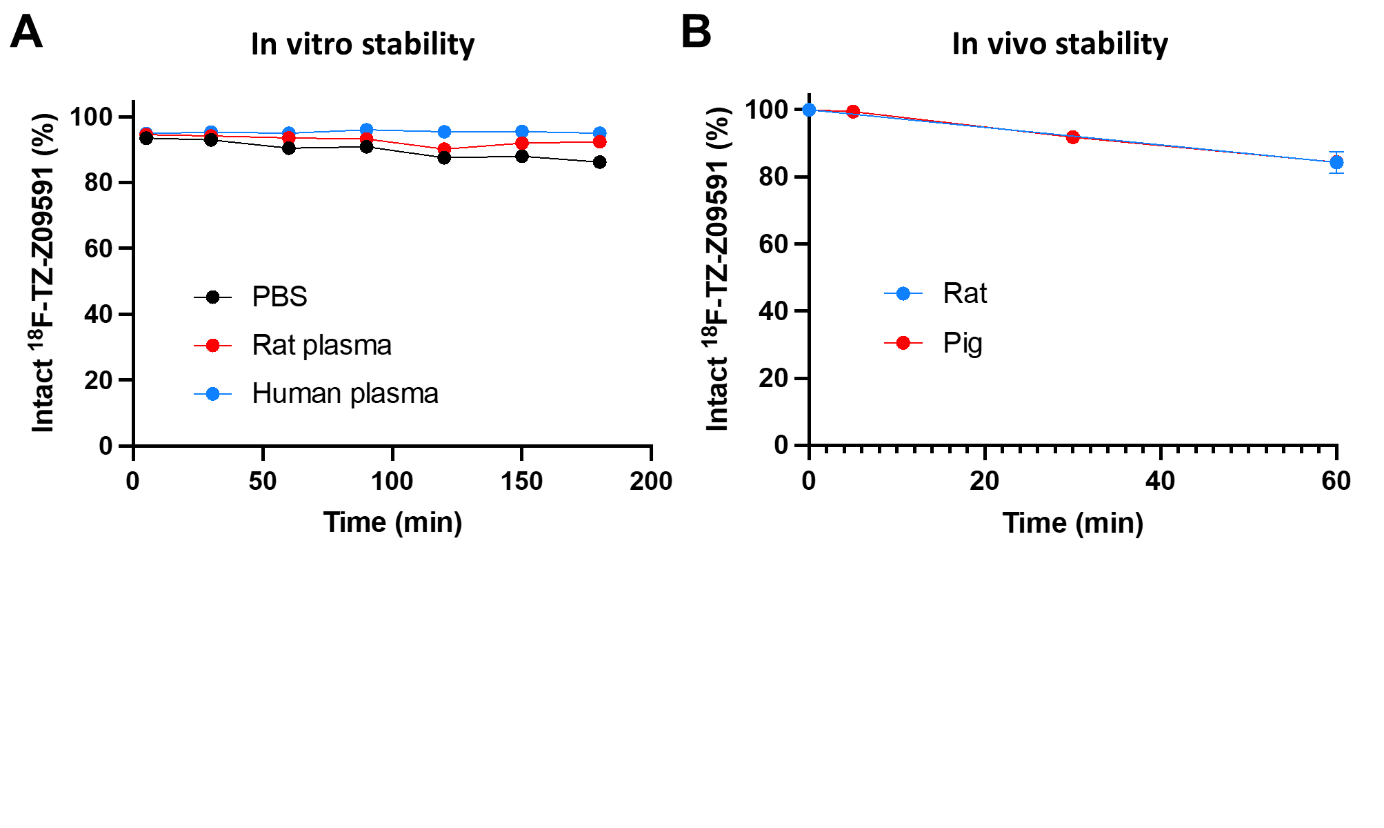


**Figure S7.** Percentage of intact [18F]TZ-Z09591 over time, measured in vitro (A) in PBS, rat plasma and pig plasma, and in vivo (B) in rat and pig.


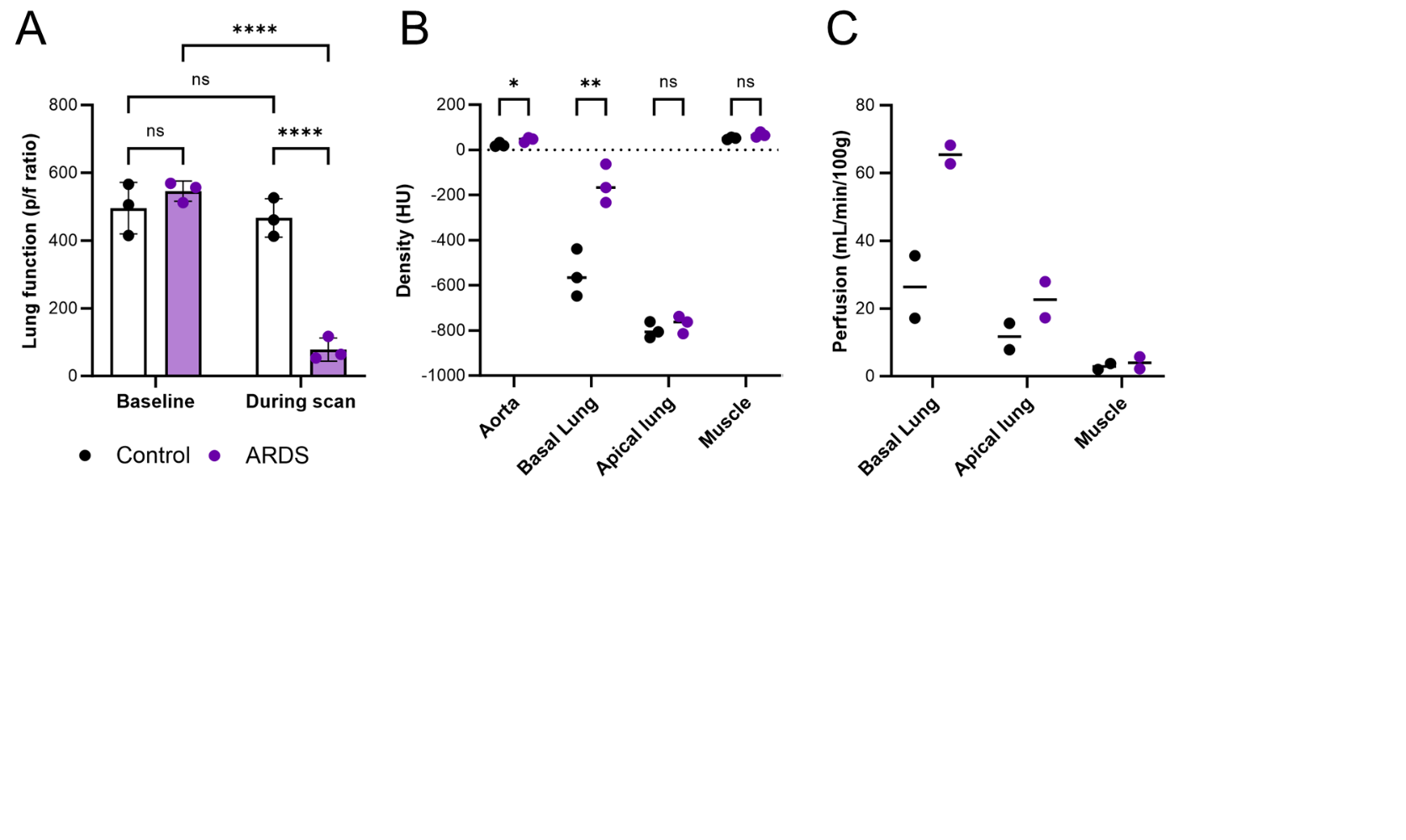


**Figure S8.** Lung function (p/f ratio) at baseline and during scan (A), lungs CT density (B) and blood perfusion (C) in control and ARDS pigs.

**Table S1.** Results of multiple unpaired t-tests with Holm-Sidak correction comparing **[¹⁸F]TZ-Z09591 uptake** in each organ of **U-87 xenografted mice** between non-blocked (n=4) and blocked (n=4) conditions (related to **Figure 2C**).

|  | **P value** | **Mean of [^18^F]TZ-Z09591 alone** | **Mean of + 1mg/kg Cys-Z09591** | **Difference** | **SE of difference** | **t ratio** | **df** | **Adjusted P Value** |
| --- | --- | --- | --- | --- | --- | --- | --- | --- |
| **Blood** | 0.028 | 1.311 | 1.069 | 0.243 | 0.084 | 2.871 | 6 | 0.0560 |
| **Lungs** | 0.003 | 1.918 | 0.781 | 1.137 | 0.243 | 4.687 | 6 | 0.0134 |
| **Liver** | 0.016 | 2.911 | 1.693 | 1.218 | 0.365 | 3.338 | 6 | 0.0462 |
| **Spleen** | <0.000001 | 5.422 | 0.932 | 4.490 | 0.191 | 23.48 | 6 | 0.000003 |
| **Kidneys** | 0.945 | 29.68 | 29.43 | 0.254 | 3.536 | 0.072 | 6 | 0.9451 |
| **Tumor** | 0.0002 | 8.579 | 2.396 | 6.183 | 0.767 | 8.056 | 6 | 0.0012 |
| **Muscle** | 0.0002 | 0.850 | 0.231 | 0.6191 | 0.078 | 7.930 | 6 | 0.0012 |
| **Bone** | 0.00008 | 1.456 | 0.467 | 0.989 | 0.106 | 9.369 | 6 | 0.0006 |

**Table S2.** Post-hoc **Tukey’s multiple comparison test** comparing **[¹⁸F]TZ-Z09591 binding** in whole lung sections of **healthy and bleomycin-treated rats** between non-blocked and blocked conditions (related to **Figure 3C**).

| **Tukey’a nultiple comparisons test** | **Mean 1** | **Mean 2** | **n1** | **n2** | **q** | **DF** | **95% CI of diff.** | **Adjusted P value** |
| --- | --- | --- | --- | --- | --- | --- | --- | --- |
| **Non blocked:Control vs. Non blocked:Bleomycin** | 6610 | 10035 | 3 | 3 | 8.289 | 8 | -5296 to -1554 | 0.0017 |
| **Non blocked:Control vs. Blocked:Control** | 6610 | 1316 | 3 | 3 | 12.82 | 8 | 3424 to 7166 | <0.0001 |
| **Non blocked:Control vs. Blocked:Bleomycin** | 6610 | 1866 | 3 | 3 | 11.48 | 8 | 2873 to 6615 | 0.0002 |
| **Non blocked:Bleomycin vs. Blocked:Control** | 10035 | 1316 | 3 | 3 | 21.11 | 8 | 6849 to 10591 | <0.0001 |
| **Non blocked:Bleomycin vs. Blocked:Bleomycin** | 10035 | 1866 | 3 | 3 | 19.77 | 8 | 6298 to 10040 | <0.0001 |
| **Blocked:Control vs. Blocked:Bleomycin** | 1316 | 1866 | 3 | 3 | 1.332 | 8 | -2422 to 1321 | 0.7841 |

**Table S3.** Post-hoc **Tukey’s multiple comparison test** comparing **[¹⁸F]TZ-Z09591 binding** in **unaffected and fibrotic lung lesions** of rats between non-blocked and blocked conditions (related to **Figure 3D**).

| **Tukey’a nultiple comparisons test** | **Mean 1** | **Mean 2** | **n1** | **n2** | **q** | **DF** | **95% CI of diff.** | **Adjusted P value** |
| --- | --- | --- | --- | --- | --- | --- | --- | --- |
| **Non blocked:Fibrotic lesions vs. Non blocked:Unaffected lung** | 14093 | 8819 | 6 | 5 | 27.33 | 17 | 4498 to 6049 | <0.0001 |
| **Non blocked:Fibrotic lesions vs. Blocked:Fibrotic lesions** | 14093 | 2448 | 6 | 5 | 60.36 | 17 | 10869 to 12421 | <0.0001 |
| **Non blocked:Fibrotic lesions vs. Blocked:Unaffected lung** | 14093 | 1591 | 6 | 5 | 64.80 | 17 | 11726 to 13277 | <0.0001 |
| **Non blocked:Unaffected lung vs. Blocked:Fibrotic lesions** | 8819 | 2448 | 5 | 5 | 31.62 | 17 | 5561 to 7181 | <0.0001 |
| **Non blocked:Unaffected lung vs. Blocked:Unaffected lung** | 8819 | 1591 | 5 | 5 | 35.87 | 17 | 6418 to 8038 | <0.0001 |
| **Blocked:Fibrotic lesions vs. Blocked:Unaffected lung** | 2448 | 1591 | 5 | 5 | 4.251 | 17 | 46.51 to 1667 | 0.0362 |

**Table S4.** Post-hoc **Tukey’s multiple comparison test** comparing **[¹⁸F]TZ-Z09591 SUVmean** from PET in **whole lungs, lung lesions, and background healthy lung regions** of **healthy and bleomycin-treated rats** (related to **Figure 4B**).

| **Tukey’a nultiple comparisons test** | **Mean 1** | **Mean 2** | **n1** | **n2** | **q** | **DF** | **95% CI of diff.** | **Adjusted P value** |
| --- | --- | --- | --- | --- | --- | --- | --- | --- |
| **Whole lungs Control vs. Whole lungs Bleomycin** | **0.387** | **0.827** | **7** | **4** | **6.123** | **15** | **-0.730 to**  **-0.147** | **0.003** |
| **Whole lungs Control vs. Lesion Bleomycin** | **0.387** | **1.014** | **7** | **4** | **8.737** | **15** | **-0.917 to**  **-0.334** | **<0.0001** |
| **Whole lungs Control vs. Background Bleomycin** | **0.387** | **0.609** | **7** | **4** | **3.080** | **15** | **-0.512 to 0.071** | **0.174** |
| **Whole lungs Bleomycin vs. Lesion Bleomycin** | **0.827** | **1.014** | **4** | **4** | **2.317** | **15** | **-0.5162 to 0.142** | **0.388** |
| **Whole lungs Bleomycin vs. Background Bleomycin** | **0.827** | **0.609** | **4** | **4** | **2.698** | **15** | **0.076 to 0.734** | **0.266** |
| **Lesion Bleomycin vs. Background Bleomycin** | **1.014** | **0.609** | **4** | **4** | **5.014** | **15** | **0.076 to 0.734** | **0.014** |

**Table S5.** Unpaired **two-tailed t-test with Welch’s correction** comparing **ex vivo [¹⁸F]TZ-Z09591 SUV** in lungs from **healthy and bleomycin-treated rats** (related to **Figure 4C**).

|  | **Weight lungs Control** | **Weight lungs Bleomycin** |
| --- | --- | --- |
| **N** | **12** | **12** |
| **mean** | **1.020** | **1.178** |
| **P value** | **0.398** | |
| **t, df** | **t=0.871, df=14.62** | |
| **95% CI** | **-0.230 to 0.547** | |

**Table S6.** Unpaired **two-tailed t-test with Welch’s correction** comparing **lung weights** between **healthy and bleomycin-treated rats** (related to **Figure 4D**).

|  | **Weight lungs Control** | **Weight lungs Bleomycin** |
| --- | --- | --- |
| **N** | **12** | **12** |
| **mean** | **1.390** | **2.811** |
| **P value** | **P<0.0001** | |
| **t, df** | **t=8.066, df=12.68** | |
| **95% CI** | **1.040 to 1.803** | |

**Table S7.** Multiple unpaired t-tests (uncorrected for multiple comparisons) comparing **Mean Standardized Uptake Value (SUV_mean_)** between **control (n=3) and ARDS pigs (n=3)** in each organ separately (related to **Figure 5B**).

|  | **P value** | **Mean of Control** | **Mean of ARDS** | **Difference** | **SE of difference** | **t ratio** | **df** |
| --- | --- | --- | --- | --- | --- | --- | --- |
| **Aorta** | 0.267 | 0.729 | 0.955 | -0.226 | 0.175 | 1.290 | 4 |
| **Ba. Lung** | 0.021 | 0.351 | 0.822 | -0.471 | 0.128 | 3.671 | 4 |
| **Ap. lung** | 0.329 | 0.147 | 0.193 | -0.046 | 0.041 | 1.110 | 4 |
| **Muscle** | 0.348 | 0.187 | 0.244 | -0.057 | 0.054 | 1.061 | 4 |

**Table S8.** Multiple unpaired t-tests (uncorrected for multiple comparisons) comparing **tracer uptake ratios (relative to the aorta)** between **control (n=3) and ARDS pigs (n=3)** in each organ separately (related to **Figure 5C**).

|  | **P value** | **Mean of Control** | **Mean of ARDS** | **Difference** | **SE of difference** | **t ratio** | **df** |
| --- | --- | --- | --- | --- | --- | --- | --- |
| **Ba. Lung** | 0.027 | 0.487 | 0.887 | -0.400 | 0.118 | 3.392 | 4 |
| **Ap. lung** | 0.922 | 0.201 | 0.204 | -0.003 | 0.025 | 0.104 | 4 |
| **Muscle** | 0.891 | 0.255 | 0.258 | -0.003 | 0.024 | 0.146 | 4 |

**Table S9.** Multiple unpaired t-tests (uncorrected for multiple comparisons) comparing **Total Distribution Volume (Vt)** between **control (n=3) and ARDS pigs (n=3)** in each organ separately (related to **Figure 5D**).

|  | **P value** | **Mean of Control** | **Mean of ARDS** | **Difference** | **SE of difference** | **t ratio** | **df** |
| --- | --- | --- | --- | --- | --- | --- | --- |
| **Ba. Lung** | 0.019 | 0.352 | 0.589 | -0.237 | 0.062 | 3.819 | 4 |
| **Ap. lung** | 0.880 | 0.142 | 0.137 | 0.005 | 0.029 | 0.162 | 4 |
| **Muscle** | 0.295 | 0.154 | 0.175 | -0.021 | 0.017 | 1.204 | 4 |

**Table S10.** Post-hoc **Tukey’s multiple comparison test** comparing **lung function (p/f ratio** of **healthy (n=3) and ARDS pigs (n=3) at baseline and during scan** (related to **Figure S6A**).

| **Tukey’a nultiple comparisons test** | **Mean 1** | **Mean 2** | **n1** | **n2** | **q** | **DF** | **95% CI of diff.** | **Adjusted P value** |
| --- | --- | --- | --- | --- | --- | --- | --- | --- |
| **Baseline:Control vs. Baseline:ARDS** | 495.7 | 546.0 | 3 | 3 | 1.658 | 8 | -187.8 to 87.16 | 0.6591 |
| **Baseline:Control vs. During scan:Control** | 495.7 | 466.7 | 3 | 3 | 0.953 | 8 | -108.5 to 166.4 | 0.9040 |
| **Baseline:Control vs. During scan:ARDS** | 495.7 | 78.04 | 3 | 3 | 13.76 | 8 | 280.1 to 555.1 | <0.0001 |
| **Baseline:ARDS vs. During scan:Control** | 546.0 | 466.7 | 3 | 3 | 2.611 | 8 | -58.21 to 216.8 | 0.3208 |
| **Baseline:ARDS vs. During scan:ARDS** | 546.0 | 78.04 | 3 | 3 | 15.41 | 8 | 330.5 to 605.4 | <0.0001 |
| **During scan:Control vs. During scan:ARDS** | 466.7 | 78.04 | 3 | 3 | 12.80 | 8 | 251.2 to 526.2 | <0.0001 |

**Table S11.** Multiple unpaired t-tests (uncorrected for multiple comparisons) comparing **different part of the lung density** between **control (n=3) and ARDS pigs (n=3)** (related to **Figure S6B**).

|  | **P value** | **Mean of Control** | **Mean of ARDS** | **Difference** | **SE of difference** | **t ratio** | **df** |
| --- | --- | --- | --- | --- | --- | --- | --- |
| **Aorta** | 0.046 | **22.31** | **44.95** | **-22.65** | 7.941 | **2.852** | 4 |
| **Ba. Lung** | 0.007 | **-550.8** | **-154.4** | **-396.3** | **78.20** | **5.068** | 4 |
| **Ap. lung** | 0.403 | **-799.7** | **-771.3** | **-28.45** | **30.46** | **0.934** | 4 |
| **Muscle** | 0.107 | 51.50 | 66.80 | -15.30 | 7.387 | 2.071 | 4 |

**Table S 12. Cell line used for the xenograft model**

| **Cells** | **RRID** | **Supplier** | **Catalog number** | **Organism** | **Tissue** | **Gender** | **Age** | **State** |
| --- | --- | --- | --- | --- | --- | --- | --- | --- |
| **U-87 MG** | **CVCL_0022** | **ATCC** | **HTB-14** | **Homo sapiens, human** | **Brain** | **Male** | **/** | **Established** |

# **References**

1. Adams, T. S. *et al.* Single-cell RNA-seq reveals ectopic and aberrant lung-resident cell populations in idiopathic pulmonary fibrosis. *Sci. Adv.* **6**, eaba1983 (2020).

2. Idiopathic Pulmonary Fibrosis Cell Atlas. https://www.ipfcellatlas.com/.

3. Betsholtzlab. Database of gene expression in adult mouse brain and lung vascular and perivascular cells. https://betsholtzlab.org/VascularSingleCells/database.html.

4. He, L. *et al.* Single-cell RNA sequencing of mouse brain and lung vascular and vessel-associated cell types. *Sci. Data* **5**, 180160 (2018).

5. Wegrzyniak, O. *et al.* Imaging of fibrogenesis in the liver by [(18)F]TZ-Z09591, an Affibody molecule targeting platelet derived growth factor receptor β. *EJNMMI Radiopharm. Chem.* **8**, 23 (2023).

6. Wegrzyniak, O. *et al.* Non-invasive PET imaging of liver fibrogenesis using a RESCA-conjugated Affibody molecule. *iScience* **27**, (2024).
